# The Association between Lead and Attention-Deficit/Hyperactivity Disorder: A Systematic Review

**DOI:** 10.3390/ijerph16030382

**Published:** 2019-01-29

**Authors:** Gabriele Donzelli, Annalaura Carducci, Agustin Llopis-Gonzalez, Marco Verani, Agustin Llopis-Morales, Lorenzo Cioni, María Morales-Suárez-Varela

**Affiliations:** 1 Department of Preventive Medicine and Public Health, Food Sciences, Toxicology and Legal Medicine, School of Pharmacy, Universitat de Valencia. Avenida Vicente Andres Estellés s/n, Burjassot, 46100 Valencia, Spain; agustin.llopis@uv.es (A.L.-G.); agustinllopis@gmail.com (A.L.-M.); Maria.M.Morales@uv.es (M.M.-S.-V.); 2 Department of Biology, University of Pisa, Via Luca Ghini, 13-56126 Pisa, Italy; annalaura.carducci@unipi.it (A.C.); marco.verani@unipi.it (M.V.); 3 Biomedical Research Consortium in Epidemiology and Public Health Network (CIBERESP), Avenida Monforte de Lemos, 3-5. Pabellón 11. Planta 0 28029 Madrid, Spain; 4 Scuola Normale Superiore, Piazza dei Cavalieri, 7-56126 Pisa, Italy; lorenzo.cioni@sns.it

**Keywords:** Lead, Attention-Deficit/Hyperactivity Disorder, ADHD, environmental pollutants, observational studies analysis, systematic review

## Abstract

The etiology of Attention-Deficit/Hyperactivity Disorder (ADHD) is complex and multifactorial. Although the development of ADHD symptoms remains to be elucidated, in recent years, epigenetic processes have emerged as candidate mechanisms. Lead is one of the most dangerous environmental pollutants, and it is suspected to be associated with ADHD. The aim of the present study was to review the epidemiological literature currently available on the relation between lead exposure and the diagnosis of ADHD. The PubMed and EMBASE databases were searched from 1 July 2018 up to 31 July 2018. The authors included observational studies (cohort, case–control and cross-sectional studies) published in English carried out on children within the last 5 years, measuring lead exposure and health outcomes related to ADHD. Seventeen studies met the inclusion criteria: 5 of these studies found no association between lead exposure and ADHD whereas the remaining 12 studies showed positive associations, even though not all of them were homogeneous in terms of exposure periods considered or ADHD diagnosis. To conclude, the evidence from the studies allowed us to establish that there is an association between lead and ADHD and that even low levels of lead raise the risk. However, there is still a lack of longitudinal studies about the relationship between lead exposure and the development of ADHD. Given the potential importance for public health, further research that includes the entire potential risk factors for ADHD in children must be encouraged.

## 1. Introduction

Attention-Deficit/Hyperactivity Disorder (ADHD) is one of the most common mental disorders affecting children [1]. ADHD also affects many adults [2]. Symptoms of ADHD are divided into two categories of inattention and hyperactivity and impulsivity that include behaviors like failure to pay close attention to details, difficulty organizing tasks and activities, excessive talking, fidgeting, or an inability to remain seated in appropriate situations [3]. 

Three types of ADHD have been identified in the Diagnostic and Statistical Manual of Mental Disorders (DSM): Combined Type, Predominantly Inattentive Type and Predominantly Hyperactive-Impulsive Type [4]. Most individuals show symptoms of both inattention and hyperactivity-impulsivity. However, there are some individuals in whom either one or the other pattern/subtype is predominant. To make an accurate diagnosis of ADHD, it is important that the appropriate subtype is indicated on the basis of the predominant symptom pattern over the past 6 months.

In the last few decades, numerous studies attempted to determine the prevalence of ADHD. However, prevalence rates for children and adolescents differ significantly among different studies, from as low as nearly 1% to as high as nearly 20% [5]. A systematic review and meta-regression analysis including 102 studies comprising 171,756 subjects from all world regions reported an ADHD/Hyperkinetic Disorder (HD) worldwide-pooled prevalence of 5.29% [6]. Another meta-analytic review covering 86 studies found that the prevalence of DSM-IV ADHD varied from 5.9% to 7.1% in children and adolescents [7]. 

While it is well-accepted that ADHD/HD is a highly heritable disorder, not all of the risk is genetic. It is estimated that between 10% and 40% of the variance associated with ADHD is likely to be accounted for by environmental factors [8]. The proposed and mostly reviewed ADHD environmental risk factors include prenatal substance exposures, heavy metal and chemical exposures, nutritional factors and lifestyle/psychosocial factors [9].

The etiology of ADHD is complex and multifactorial. The importance of both environmental risk factors and genetic factors has been indicated in several epidemiological studies. The development of ADHD symptoms remains to be elucidated, but in recent years, epigenetic processes have emerged as candidate mechanisms [10]. Considering that the genetic factors are non-modifiable risk factors in the short run, in order to decrease the risk of ADHD, it is necessary to reduce the exposure to environmental risk factors.

As to the environmental risk factors, the authors focused on the pollution from lead. Lead is a naturally occurring bluish-gray metal present in small amounts in the earth’s crust [11]. Its widespread use has resulted in extensive environmental contamination, human exposure and significant public health problems of global dimensions [12].

Lead is one of the most dangerous environmental pollutants. The WHO has identified lead as one of ten chemicals of major public health concern that require action by the Member States in order to protect the health of workers, children and women of reproductive age [13]. Lead exposure is responsible for 540,000 deaths and 13.9 million years lost to disability and death due to long-term effects on health. Developing regions are paying the highest price in terms of the burden of this disease [14]. In environmental exposure, the various polluting agents rarely exist in isolation so the combined exposure to lead and other pollutants or risk factors may result in more severe outcomes [15,16].

Patterns and sources of exposure to lead, prevalence rates of lead poisoning and the severity of outcomes vary greatly from country to country and from place to place within countries [17]. People are exposed to lead from different sources and through different pathways (such as air, food, water, dust and soil). Lead-based paint is the most widespread and dangerous high-dose source of lead exposure for young children [18]. Lead also occurs in drinking water through leaching from lead-containing pipes, faucets and solder frequently found in the plumbing of older buildings. Lead in lead-glazed food containers may contaminate water, food and beverage. Lead may also be found in and around workplaces; in some commercial products (e.g., imported jewelry and candies; children's toys; cosmetics; and folk, traditional or home remedies); in second- and third-hand tobacco smoke exposures (SHS and THS); and in soil, air and water near the sites of historic or ongoing mining operations or smelters [19].

Lead exposure produces a variety of adverse health effects in children. It may cause intellectual, behavioral or motor function deficits as well as hand–eye coordination and reaction time problems and a lowered performance on intelligence tests [20]. Long-term lead exposure can also damage the kidneys and nervous system and increase the risk of high blood pressure in adults [21]. Furthermore, maternal exposure to high levels of lead during pregnancy may be associated with increased incidences of miscarriage, stillbirth, premature birth and low birth weight [22,23]. Currently, there is no safe threshold for lead exposure, but it is known that, as lead exposure increases, the range and severity of symptoms and effects also increases [24]. Scientific evidence has shown that even low levels of lead exposure, BLLs >5 ug/dL, may cause a decrease in intelligence and an increase of behavioral and learning difficulties in children [25].

Not disregarding the genetic factors, the authors focused only on the environmental pollution from lead. Therefore, the aim of this study has been to perform a systematic review in order to explore and analyze the existing literature for the potential relationships between lead exposure and ADHD so to have a deeper and up-to-date understanding of the effects of this pollutant on the mental health of children.

## 2. Methods

### 2.1. Study Identification and Eligibility Criteria

A preliminary search performed to assess the prevalence of other systematic reviews covering the possible association between lead and hyperactivity disorder yielded 2 relevant articles [26,27] which the findings of have been considered when discussing the conclusions. The databases on which the query was performed in order to identify the publications eligible for inclusion in the review were EMBASE and MEDLINE (accessed from PubMed). The literature searches were conducted using the keywords “environmental”, “pollution”, “lead” and “hyperactivity disorder” in the following query:

((“environmental” OR “pollution” OR “lead”) AND “hyperactivity disorder”)).

**Figure 1:**
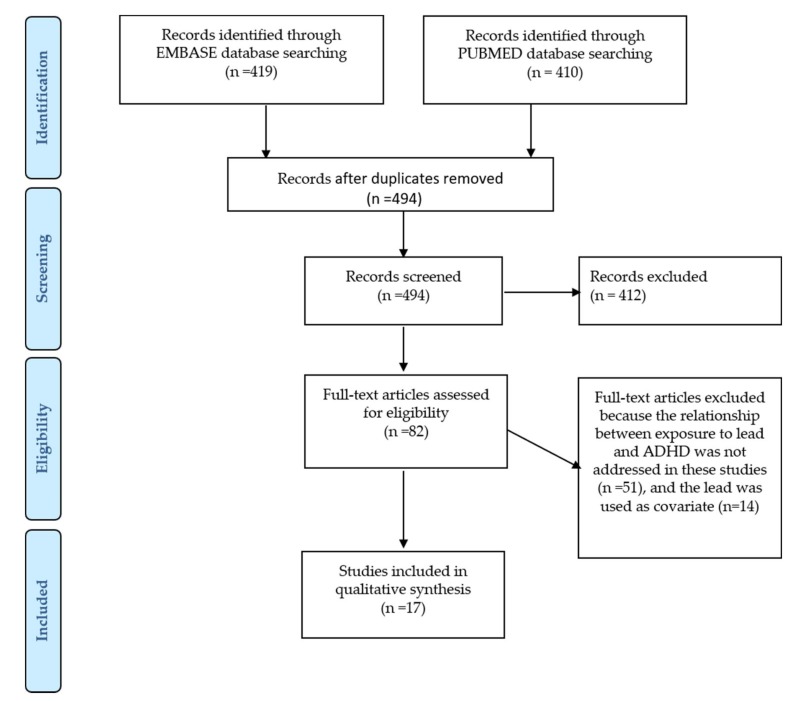
A PRISMA flow diagram for ADHD: Attention-Deficit/Hyperactivity Disorder.

Moreover, the authors have used the following research filters: articles published from 1 July 2013 to 30 June 2018, the exclusion of animal studies and publication in the English language. Figure 1 shows the search strategy followed for this review. A total of 829 articles were identified. An initial screening identified 82 candidate studies. The initial screening of the studies was performed using the information available in both the titles and the abstracts. These potentially relevant studies were retrieved in full text and assessed for eligibility according to the following criteria:

1. the inclusion of humans as study subjects without restriction on the demographic characteristics of the population;
2. the conduction of an exposure assessment to lead during pregnancy or early childhood; and
3. the inclusion of measures of hyperactivity disorder symptoms or diagnosis.

The publications were included in the analysis only if they met all the eligibility criteria. After a full assessment of the potentially relevant studies, 17 were proposed to be included in the present systematic review.

When writing this paper, the authors fully considered the methodological norms established for the publication of systematic reviews and the Preferred Reporting Items for Systematic Reviews and Meta-Analyses (PRISMA) recommendations [28,29].

### 2.2. Internal Validity

To further assess the chosen articles and guide the evaluation of the data included in them, the authors classified the publications using the scale proposed by the Scottish Intercollegiate Guidelines Network [30] for establishing the levels of evidence (Table 1) and grades of recommendation (Table 2). The aim of the Scottish Intercollegiate Guidelines Network (SIGN) system is to ensure that the grade of the internal and external validity of a study is robustly assessed and leads to the final grade for a recommendation. The methodology behind this system is based on a set of variables that recognize key factors, especially biasing and confounding factors, that can influence the quality of a study or its conclusions. The SIGN system emphasizes the aspects of study design (systematic reviews of Randomized Controlled Trials or RCTs and meta-analyses, RCTs, cohort studies, case–control studies, non-analytic studies and expert opinion) which can lead to biased results, and importantly, the SIGN system also identifies the direction of that bias. Though this methodology clearly gives the gold standard to RCTs, it is recognized that non-randomized studies can strengthen or put in doubt the results of RCTs [31]. The evidence is classified by its epistemological strength, and only the strongest evidence gives way to strong recommendations while the weaker evidences can only give rise to weak recommendations. The SIGN scale of the level of evidence proposes that the study design and the risk of bias are used to assess the level of evidence or the quality of the scientific evidence provided. In order to rate the study design, the numbers “1”, “2”, “3” and “4” are used, and while grading the criteria of “++”, “+” or “−“ are used to represent the assessed risk of bias. Based on this assessment of the quality of the evidence in the articles, the strength of the associated recommendations is classified according to “A”, “B”, “C” and “D” grades in order from the best to the worst.

Such a graded scale has been derived from the principles of evidence-based medicine (EBM). EBM is an approach that assures the use of the most up-to-date, reliable and scientifically solid evidence available in making decisions about a particular situation being studied (Sackett, 1997) [32]. Ethical or other limitations can affect the quality or rating of the current best evidence available, taking into account the defined area of study. Some limitations are, at times, insurmountable; however, these must not be seen as detrimental to the study, as the example of the link between ADHD and lead exposure does not lend itself to randomized clinical trials. Ethical constraints, moreover, limit the current best available evidence to case–control or cohort-type studies. This proves to be a challenge when the authors try to establish an association between ADHD and lead since it means that any included study can at most receive a 2++B rating. From all this, the authors derive that the recommendations extracted from the studies that the authors present in this review can, at the most, be classified as moderately strong or 2++B. However, given that the principles of EBM have been followed correctly, the conclusions of this review are valid as they can be derived from the best currently available evidence.

All of the studies that the authors included in the present review are either case–control or cohort studies and, therefore, can only be scored as level 2. The assessed risk of bias and the degree of probability that the relationship is causally represented place most of the studies in the 2+ category given their results (as shown in Table 3). This prevalence limits the strength of the associated recommendations to grades C. This is true also if the authors consider the target population and the consistency of the results in each of the studies so that most of them end up categorized as having a grade of recommendation C. 

We also focused on the internal validity of the studies analyzed. The factors that influencing internal validity that were considered are:

1. a sufficiently large sample size;
2. the specification of the inclusion and assessment criteria;
3. an accurate diagnosis of ADHD and lead exposure assessment; and
4. an adjustment for the confounding variables.

The classification according to the SIGN scale and the assessment of the internal validity were performed mainly by one author with frequent consultations with a second author, and once a consensus among the two was reached, the classification was given to the other authors for revision and approval. No notable disagreements arose among the authors at this point.

### 2.3. Data Extraction

During the analysis of the results from the different studies, the authors noticed how these had been expressed either in a non-homogenous or in a non-standardized manner. Therefore, the authors standardized and presented the results in a single integrated scale in order to avoid any possible confusion. However, the available data and the methodologies utilized in the various studies restricted the standardization to a limited number of the studies (7 out 17 articles).

## 3. Results

### 3.1. Characteristics of the Studies

**Figure 2:**
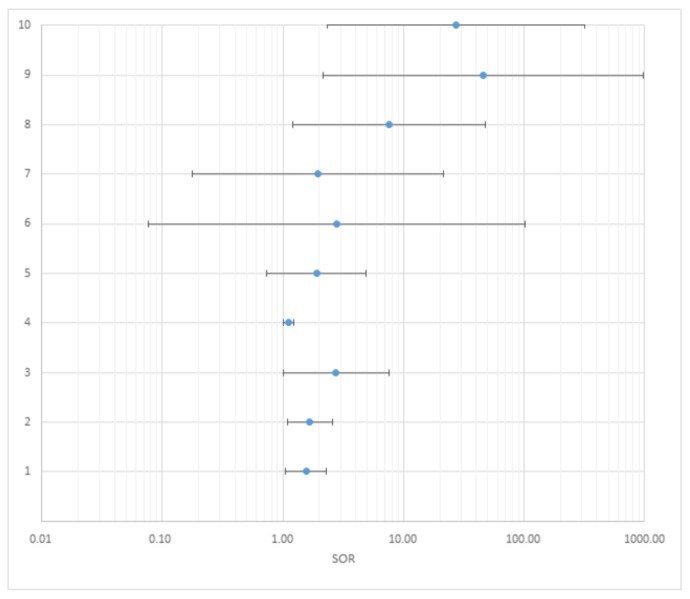
The standardized odds ratios (per 5 µg dL).

The chosen studies were analyzed according to the following characteristics: location, sample size, birth years or range of age, study design, ADHD measurement criteria, exposure measurement, results, level of evidence and grade of recommendation. Table 3 summarizes the characteristics of the studies. Table 4 and Figure 2 show the Odd Ratios (ORs) standardized to 5 µg/dL for 7 out of the 17 articles included.

### 3.2. Study Design and Population

Two cross-sectional studies, 5 cohort studies and 10 case–control studies drawn from 9 different countries are included in this study. Sample sizes ranged from 117 [37] to 2195 [36] summing up to a total of 8940 participants.

Three studies used a Taiwanese population [41,44,45] and four South Korean populations [36,42,43,48]. Within the studies that used European populations, one used a German population [37], one a Spanish population [38] and one a Belgian population [39]. The other studies used an American population [35,46,48], a Chinese population [34,40], a Turkish population [49] and a Mexican population [33].

### 3.3. Measurement of Lead Exposure

Fourteen out of 17 studies have determined the levels of lead in blood (BLLs). Blood samples were obtained from each child via venipuncture in the arm. Two of these studies, References [35,39], also collected and analyzed the cord blood lead levels of the mothers. One each of the cohort [38] and case–control [41] studies collected urine samples and analyzed them by inductively coupled plasma mass spectrometry (ICP-MS). Another study [46] collected molar teeth and longitudinally sectioned them with a diamond blade on an Isomet low-speed saw (Buehler, Lake Bluff, IL, USA). Lead concentrations were determined through Inductively Coupled Plasma-Optical Emission Spectroscopy (ICP-OES) with a sensitivity limit of 0.2 µg/L.

### 3.4. Cross-Sectional Studies

Huang and coauthors [33] have shown that blood lead levels among Mexican children with low exposure (≤5 μg/dL) were positively associated with hyperactive/impulsive behaviors but not with inattentiveness. Zhang and coauthors [34] investigated the ADHD status among preschool-aged children in Guiyu, an electronic waste (e-waste) recycling town in Guangdong, China. The study showed that children with high BLLs (≥10 µg/dL) had a 2.4 times higher risk of ADHD than those with low BLLs (<10 µg/dL). 

### 3.5. Cohort Studies

Ji and coauthors [35] analyzed the data from 1479 mother–infant pairs (299 ADHD and 1180 neurotypical) in the Boston Birth Cohort. Lead levels were analyzed both as a binary variable and as 3 categories. In the first case, children with 5–10 μg/dL lead levels compared to those with less than 5 μg/dL had 66% increased odds of having an ADHD diagnosis. In the second case, children with 2–4 μg/dL and 5–10 μg/dL lead levels compared with those with less than 2 μg/dL had an OR of 1.08 (95% CI, 0.81–1.44) and 1.73 (95% CI, 1.09–2.73), respectively. Choi and coauthors [46] showed that, after an adjustment for potential confounders, ADHD developed more frequently in children with blood lead levels >2.17 μg/dL (highest quartile) compared with those with blood lead levels <2.17 μg/dL. Neugebauer and coauthors [37] showed that lead levels were positively correlated with ADHD. Impulsivity significantly increased by 20% with each doubling of blood lead concentrations (geometric mean ratio: 1.20; 95% CI: 1.08–1.33). On the Overall ADHD scale, the increase was approximately 9% per doubling of the lead concentration (geometric mean ratio: 1.09; 95% CI: 1.01–1.17). Forns and coauthors [38] analyzed the data from a population-based cohort established in the city of Sabadell (Barcelona, Catalonia, Spain). In this study, no association was found between ADHD symptomatology (inattention and hyperactivity) and lead levels. Sioen and coauthors [39] found that in all children, the prenatal lead exposure was significantly associated with hyperactivity at the ages of 7–8 years.

### 3.6. Case–Control Studies

Yang and coauthors [40] investigated the trace element status of lead in children with ADHD and compared them with normal controls. The analyses were performed according to different age groups: childhood (from 6 to 11 years of age) and adolescence (from 12 to 16 years of age). No significant relationship was indicated between lead and ADHD symptoms. Lee and coauthors [41] carried out a case–control study in order to investigate the possible differences in the urinary levels of lead between patients with different ADHD subtypes and the healthy controls. They found that lead levels were positively correlated with the inattention and hyperactivity/impulsivity symptoms. Joo and coauthors [42] found that BLLs were significantly associated with inattention when the model was adjusted for postnatal second-hand smoke exposure (OR = 1.63; 95% CI = 1.03–2.58). Park and coauthors [43] revealed that the children with blood lead concentrations above 2.30 μg/dL were at a 2.5-fold (95 % CI: 1.09–5.87, *p* < 0.05) greater risk of ADHD. Yu and coauthors [44] found no significant difference in BLLs between children with and without ADHD. Yu and coauthors [45] showed that there was no significant difference in BLLs between children with and without ADHD (*p* = 0.15). Chan and coauthors [46] found no significant association between ADHD symptoms and the concentration of lead in teeth. Hong and coauthors [47] found that the association of blood lead with higher impulsivity was robust to the adjustment for a variety of covariates. However, blood lead levels were not significantly associated with inattention in the adjusted models. Kim and coauthors [48] showed that blood lead concentration was not related to ADHD in the unadjusted analysis, but after considering the covariates, high lead concentrations were associated with a higher risk of ADHD. The pattern was similar using categorical blood lead (≥2 or ≥3 µg/dL). Dikme and coauthors [49] found no significant difference between the patients and control groups in terms of lead levels (*p* = 0.575). 

## 4. Discussion

ADHD is a persistent neurodevelopmental disorder that affects 5% of children and adolescents and 2.5% of adults worldwide [50]. ADHD heritability, estimated from 60% to 80%, highlights the considerable role of environmental factors in the disorder susceptibility [9]. It is accepted that both biological and environmental factors can contribute to the development of ADHD. Several studies showed that human exposures to environmental pollutants can represent a risk factor for ADHD. For example, the exposure to heavy metals; dietary factors [51]; and the environmental exposure to dangerous chemicals such as bisphenol A [52], polycyclic aromatic hydrocarbons [53] and pesticides [54] may contribute to ADHD. 

Currently, no safe blood lead level in children has been identified. The Center for Disease Control Advisory Committee on Childhood Lead Poisoning Prevention recommended that blood lead levels at or above 5 µg/dL are sufficient to initiate public health actions [19]. However, a recent meta-analysis [27] has indicated that even blood lead levels <3 μg/dL may be associated with ADHD symptoms in children. These findings emphasize that there is no safe blood lead threshold and that if public actions focus only on the reduction of the exposure of children to high lead levels, they fail to protect children with lower levels that represent the larger group.

Another systematic review [26] has examined the literature on the role of lead exposure in children with ADHD symptoms. However, in this other review, there are included articles up to May 2014 and only studies in which lead exposure was examined using blood samples have been considered. The results highlighted that in 16 out of the 18 studies considered, there was a significant association between BLLs of less than 10μg/dL in children and at least one type of ADHD [26]. Our systematic review expands the body of knowledge about this subject because it includes more recent literature (articles published from 1 July 2013 to 30 June 2018) and studies which considered urine samples and teeth. In the same way, the majority of the studies the authors considered have revealed a significant association between environmental lead exposure and ADHD.

### 4.1. Summary of the Evidence

The results of the present study revealed that in 12 out of the 17 studies, a significant association was found between lead exposure and one of the types of ADHD. One thing that must be considered is that 4 of the 5 studies without any significant association were classified as 2- in the scale used to individually evaluate the levels of evidence and, owing to their high risk of bias, these should not be used in compiling recommendations. Furthermore, the remaining study which did not find any association considered the level of lead in urine samples. This fact can represent a bias due to an individual variation in concentration and since urinary lead levels are less sensitive in the lower range of exposure, for example, at blood lead concentrations lower than 10 µg/dL [55]. However, the current results must be interpreted with caution owing to the presence of a high heterogeneity.

### 4.2. Strengths and Limitations of the Current Review

The searches may have failed to retrieve all the relevant publications concerning the association between ADHD and lead owing to the fact that the field of analysis was restricted to studies published in English available through the Pub Med and EMBASE databases. This review, like any other review about observational data, may suffer biases related to the publicity of the studies since it is believed that studies with significant positive results are more widely distributed than those without significant results or with negative ones [56].

#### Conceptual Constraints

The authors have included the keywords “environmental” and “pollution” to collect also the studies where the keyword “lead” was not present in the title and abstract. However, in the publications retrieved, the keywords “environmental” and “pollution” carry a variety of meanings as there are no standardized definitions of these concepts. In this review, the authors only focused on environmental lead pollution and its impact on children's health. This represents a constraint to the research because it limits the number of studies included in this review.

### 4.3. Strengths and Limitations of the Studies Included in the Review

#### 4.3.1. ADHD Diagnosis

The use of a medical diagnosis of ADHD by a physician based on the Diagnostic and Statistical Manual of Mental Disorders reduces the likelihood of a misdiagnosis. However, the majority of the reviewed studies reports data from parents’ responses or teachers’ responses to behavior checklists (e.g., SNAP-IV [57]) that vary from one study to another, and this fact may have produced misdiagnosis or biases. Furthermore, some studies did not consider the separation of ADHD inattention and hyperactivity-impulsivity symptoms.

#### 4.3.2. Observation and Exposure Periods

The observation and exposure periods used in the studies reviewed are not homogenous. If the studies are performed at too early an age, the rate of detection or misdiagnosis can possibly be significant so to alter the results.

#### 4.3.3. Lead Exposure Assessment

This review has also included studies in which lead exposure was examined using urine and teeth. However, the main limitation of using urine samples is the individual variation. In addition, urinary lead is less sensitive in the lower range of exposures (i.e., <10 µg/dL), and for this reason, the authors believe that the quantification of lead in blood is more appropriate. Regarding the use of teeth, the authors should take into consideration that there is presently no well-defined low, medium or high concentration levels for environmental risk factors such as lead. All concentrations measured can only be used in relation to other samples in the study when analyzing teeth.

#### 4.3.4. Measures of Association

The association between the lead exposure and the risk of having ADHD was calculated following different approaches. Logistic regression models have been used in the majority of studies to obtain the adjusted odds ratios. However, lead levels were analyzed as continuous, binary and/or categorical variables on the basis of different cutoff points obtained from previous studies and the Centers for Disease Control and Prevention (CDC) guidelines. The remaining studies have used the Mann–Whitney U test or Spearman’s correlation test to analyze the relationships between lead levels and ADHD diagnosis.

#### 4.3.5. Cofounders

The existence of confounding variables in a study may make it difficult to establish a clear causal link between the exposure and outcome unless appropriate methods are used to adjust for the effect of the confounders [58]. Most of the studies examined in this review considered, indeed, the confounding variables such as maternal marital status, age, educational years, socioeconomic status, maternal smoking during pregnancy, child’s age at behavioral testing, sex, birth weight, paternal educational years, etc. However, not all the reviewed studies considered the same potential confounding variables, and this could be a source of information bias in this review. Moreover, five of the articles included in this review and classified with the level 2- did not consider any confounding variables.

## 5. Conclusions

Based on the results of this review, additional data is needed to fully ascertain the nature of the relationship between lead exposure and ADHD. Future research should consider the influence of all potentially confounding variables and also use a standardized method of ADHD diagnosis. Future studies should be focused on lead exposure of the mothers during late pregnancy and the first years of life of the children. The combined exposure to multiple chemicals or risk factors should also be evaluated together with the influence of genetic factors.

## Figures and Tables

**Table 1:** The levels of evidence [30].

	Levels of Evidence	
1	1++	High-quality meta-analyses, systematic reviews of RCTs, or RCTs with a very low risk of bias
	1+	Well-conducted meta-analyses, systematic reviews of RCTs, or RCTswith a low risk of bias
	1-	Meta-analyses, systematic reviews, or RCTs with a high risk of bias
2	2++	High-quality systematic reviews of case–control or cohort studies High-quality case–control or cohort studies with a very low risk of confounding or bias and a high probability that the relationship is causal
	2+	Well-conducted case–control or cohort studies with a low risk of confounding or bias and a moderate probability that the relationship is causal
	2−	Case–control or cohort studies with a high risk of confounding or bias and a significant risk that the relationship is not causal
3		Non-analytic studies, e.g., case reports, case series
4		Expert opinion

Abbreviations: SIGN: Scottish Intercollegiate Guidelines Network (2008); LE: levels of evidence; RCT: randomized and controlled trials.

**Table 2:** The grades of recommendation [30].

Grades of Recommendation	
A	At least one meta-analysis, systematic review, or RCT rated as 1++, and directly applicable to the target population A body of evidence consisting principally of studies rated as 1+, directly applicable to the target population, and demonstrating the overall consistency of results
B	A body of evidence including studies rated as 2++, directly applicable to the target population, and demonstrating the overall consistency of results Extrapolated evidence from studies rated as 1++ or 1+
C	A body of evidence including studies rated as 2+, directly applicable to the target population, and demonstrating the overall consistency of results Extrapolated evidence from studies rated as 2++
D	Evidence level 3 or 4 Extrapolated evidence from studies rated as 2+

Abbreviations: SIGN: Scottish Intercollegiate Guidelines Network (2008). GR: Grade of Recommendation; RCT: Randomized and Controlled Trials.

**Table 3:** The studies on lead exposure and ADHD.

Citation	Location	Sample Size (Birth Years)	Study Design	ADHD Symptom Measured	Exposure Measurement	Results	LE	GR
Huang et al., 2016 [33]	Mexico	4126–13 years	Cross-sectional	Inattention Hyperactivity/Impulsivity Combined ADHD	BLLs	BLLs among children with low exposure (≤5 μg/dL) was positively associated with hyperactive/impulsive behaviors but not with inattentiveness.	2+	C
Zhang et al., 2015 [34]	China	2433–7 years	Cross-sectional	Inattention Hyperactivity/Impulsivity Combined ADHD	BLLs	The children with high BLLs (≥10 µg/dL) had a 2.4 times higher risk of ADHD than the children with low BLLs.	2+	C
Ji et al., 2018 [35]	Boston	1479 mother–infant pairs (299/1176)	Birth cohort 1998–2013	Combined ADHD	BLLs	Children with 5–10 μg/dL lead levels had 66% increased odds of having an ADHD diagnosis as compared with children with less than 5 μg/dL lead levels.	2+	C
Choi et al., 2016 [36]	South Korea	2195	Birth cohort 2005–2010	Combined ADHD	BLLs	Relative risk for ADHD symptoms was 1.552 in children with blood lead levels > 2.17 μg/dL (highest quartile) compared with those with blood lead levels of ≤2.17 μg/dL.	2+	C
Neugebauer et al., 2014 [37]	Germany	117	Birth cohort 2000–2002	Inattention Hyperactivity/Impulsivity Combined ADHD	BLLs	Lead exposure was positively associated with ADHD. Hyperactivity/Impulsivity and Combined ADHD significantly increased by 20% and 9% per each doubling of BLLs, respectively.	2+	C
Forns et al., 2014 [38]	Spain	385	Birth cohort 2004–2006	Inattention Hyperactivity/Impulsivity	Urine sample	No statistically significant associations between lead and ADHD.	2+	C
Sioen et al., 2013 [39]	Belgium	270	Birth cohort 2002–2003	Combined ADHD	BLLs	Doubling the prenatal lead exposure is associated with an odds ratio for hyperactivity of 3.43.	2+	C
Yang et al., 2018 [40]	China	421/395 6–16 years	Case-control	Combined ADHD	BLLs	No statistically significant associations between lead and ADHD.	2−	
Lee et al., 2018 [41]	Taiwan	76/46 < 10 years	Case-control	Inattention Hyperactivity/Impulsivity	Urine sample	BLLs were positively correlated with inattention and hyperactivity/impulsivity symptoms (*p* < 0.05).	2−	
Joo et al., 2017 [42]	South Korea	214/2146–10 years	Case-control	Inattention Hyperactivity/Impulsivity Combined ADHD	BLLs	Exposure to low BLLs (geometric mean = 1.65 μg/dL) was associated with inattention symptoms but not with hyperactivity/impulsivity.	2+	C
Park et al., 2016 [43]	South Korea	114/114	Case-control	Inattention Hyperactivity/Impulsivity Combined ADHD	BLLs	Children with a blood lead concentration > 2.30 μg/dL had a 2.5 times higher risk of ADHD.	2+	C
Yu et al., 2016 (a) [44]	Taiwan	173/159	Case-control	Combined ADHD	BLLs	No statistically significant associations between lead and ADHD.	2−	
Yu et al., 2016 (b) [45]	Taiwan	97/1104–15 years	Case-control	Combined ADHD	BLLs	No statistically significant associations between lead and ADHD.	2−	
Chan et al., 2015 [46]	USA	266 11–13 years	Case-control	Inattention Hyperactivity/Impulsivity Combined ADHD	Analysis of teeth	BLLs were significantly associated with increased incidents of Hyperactivity/Impulsivity and Inattention.	2+	C
Hong et al., 2015 [47]	South Korea	10018–11 years	Case-control	Inattention Hyperactivity/Impulsivity Combined ADHD	BLLs	BLLs were significantly associated with parent and teacher ratings for Hyperactivity/Impulsivity but not with Inattention.	2+	C
Kim et al., 2013 [48]	USA	71/58 3–7 years	Case-control	Combined ADHD	BLLs	High BLLs were associated with a higher risk of ADHD.	2+	C
Dikme et al., 2013 [49]	Turkey	59/591.6–16 years	Case-control	Combined ADHD	BLLs	No statistically significant associations between lead and ADHD.	2−	

Abbreviations: BLLs, Blood Lead Levels.

**Table 4:** A summary of the results.

Citation	N°	Adjusted ORs			Standardized ORs		
		OR	Lower 95% CI	Upper 95% CI	OR	Lower 95% CI	Upper 95% CI
Zhang et al., 2015 [34]							
—binary: cutoff 10 µg/dℓ							
All ADHD	1	2.4	1.1	5.2	1.55	1.05	2.28
Ji et al., 2018 [35]							
—binary: cutoff 5 µg/dL							
All ADHD	2	1.66	1.08	2.56	1.66	1.08	2.56
Choi et al., 2016 [36]							
—binary: cutoff 2.17 µg/dℓ							
All ADHD	3	1.552	1.002	2.403	2.753	1.005	7.539
Neugebauer et al., 2014 [37]							
—doubling of exposure concentrations							
All ADHD	4	1.09	1.01	1.17	1.12	1.01	1.22
Joo et al., 2017 [42]							
—binary: cutoff 1.90 µg/dℓ							
All ADHD	5	1.28	0.89	1.83	1.91	0.74	4.91
Park et al., 2016 [43]							
—categorical							
All ADHD (1.13–1.71 μg/dℓ)	6	1.26	0.56	2.84	2.78	0.08	101.35
All ADHD (1.72–2.29 μg/dℓ)	7	1.26	0.55	2.87	1.96	0.18	21.43
All ADHD (2.30–5.35 μg/dℓ)	8	2.54	1.09	5.94	7.59	1.21	48.10
Kim et al., 2013 [48]							
—categorical							
All ADHD (>2 μg/dℓ)	9	4.63	1.36	15.72	46.13	2.16	979.79
All ADHD (>3 μg/dℓ)	10	7.25	1.66	31.67	27.16	2.33	317.02

The column N° refers to Figure 2.
